# Bersaldegenin-1,3,5-orthoacetate induces caspase-independent cell death, DNA damage and cell cycle arrest in human cervical cancer HeLa cells

**DOI:** 10.1080/13880209.2020.1866025

**Published:** 2021-01-06

**Authors:** Justyna Stefanowicz-Hajduk, Magdalena Gucwa, Barbara Moniuszko-Szajwaj, Anna Stochmal, Anna Kawiak, J. Renata Ochocka

**Affiliations:** a Department of Biology and Pharmaceutical Botany, Medical University of Gdańsk, Gdańsk, Poland; b Department of Biochemistry and Crop Quality, Institute of Soil Science and Plant Cultivation, State Research Institute, Puławy, Poland; c Laboratory of Plant Protection and Biotechnology, Intercollegiate Faculty of Biotechnology, University of Gdańsk and Medical University of Gdańsk, Gdańsk, Poland

**Keywords:** Bufadienolides, Kalanchoe, cytotoxicity, flow cytometry, RTCA, ROS, MMP

## Abstract

**Context:**

Bufadienolide compounds occur in many plants and animal species and have strong cardiac and anti-inflammatory properties. The compounds have been recently investigated for cytotoxic and antitumor activity.

**Objective:**

The cytotoxic effect of bersaldegenin-1,3,5-orthoacetate **–** a bufadienolide steroid occuring in plants from *Kalanchoe* genus (Crassulaceae), was evaluated with cervical cancer HeLa cells *in vitro*.

**Materials and methods:**

The cytotoxic activity of the compound (at 0.1–20.0 μg/mL) on the cells was determined by Real-Time Cell Analysis (RTCA) system for 24 h. The estimation of cell cycle arrest, reactive oxygen species (ROS) production, reduction of mitochondrial membrane potential (MMP), and caspases-3/7/9 activity in the HeLa cells treated with the compound was done by flow cytometry and luminometric technique. DNA damage in the cells was estimated by immunofluorescence staining and the comet assay with etoposide as a positive control.

**Results:**

The compound had strong effect on the cells (IC_50_ = 0.55 μg/mL) by the suppression of HeLa cells proliferation in G2/M phase of cell cycle and induction of cell death through double-stranded DNA damage and reactive oxygen species overproduction. Furthermore, we did not observe an increase in the activity of caspase-3/7/9 in the treated cells as well as a decrease in cellular mitochondrial membrane potential. Gene expression analysis revealed the overexpression of NF-Kappa-B inhibitors genes (>2-fold higher than control) in the treated cells.

**Conclusions:**

Bersaldegenin-1,3,5-orthoacetate induces cell cycle arrest and caspase-independent cell death through double-stranded DNA damage. These results are an important step in further studies on cell death signalling pathways induced by bufadienolides.

## Introduction

Bufadienolides are C-24 steroids which a have six-membered lactone ring attached at the C-17β position of the perhydrophenanthrene core. The name of this group of compounds comes from genus *Bufo* (the toad) which contains bufadienolides (Kamboj et al. 2013). These compounds were identified in species like *Bufo marinus* L. (Bufonidae) (Matsukawa et al. 1997), *B. viridis* Laurenti (Uasnova et al. 2002), *B. gargarizans* Cantor (Tian et al. 2010), *B. rubescens* Lutz (Cunha Filho et al. 2005), *Fusarium poae* (Peck) Wollenw. (Nectriaceae), *F. sporotrichioides* Sherb. (Morishita et al. 1992), *Photinus* (Lampyridae), and *Rhabdophis* (Colubridae) (Kamboj et al. 2013). In addition to animals, many plant species also contain bufadienolides. They were identified in *Kalanchoe* (Crassulaceae), *Tylecodon* (Crassulaceae), *Helleborus* (Ranunculaceae), *Scilla* (Hyacinthaceae), *Cotyledon* (Crassulaceae), *Urginea* (Hyacinthaceae), *Mimosa* (Fabaceae), *Millettia* (Fabaceae), and *Drimia* genus (Hyacinthaceae) (Stoll et al. 1933; Wagner et al. 1985; Steyn et al. 1986; Botha et al. 1998; Pohl et al. 2001; Watanabe et al. 2003; Goel & Ram 2009).

The compounds have been used from ancient times as medicines in cardiac dysfunction due to their strong effect on the heart. Nowadays, they are well-known as inhibitors of the Na^+^/K^+^-ATPase pump and cause increase in the contractile force of the heart (Kamboj et al. 2013). Furthermore, they alter myocardial ion balance and increase intracellular Ca^2+^ concentration (Melero et al. 2000; Schoner & Scheiner-Bobis 2007; Kolodziejczyk-Czepas & Stochmal 2017). Among other pharmacological properties, bufadienolides have also antiviral, antibacterial, insecticidal, anti-angiogenic, anti-inflammatory and immunomodulatory activities (Kamboj et al. 2013). Recently, the compounds have been investigated for cytotoxic and anticancer properties in normal and cancer cell lines (Gao et al. 2011; Han et al. 2016). The results indicate that this group of compounds causes significant inhibition of cell growth, proliferation and induction of cell death. For example, Iguchi et al. isolated bufadienolides from *Helleborus foetidus* L. and showed their cytotoxicity against leukaemia (HL-60) and lung cancer (A549) cell lines (Iguchi et al. 2020). Proscillaridin A revealed antitumor effects on glioblastoma cells, but not on normal cells – astrocytes and oligodendrocytes (Berges et al. 2018). Next, cinobufagin was tested on human breast cancer MCF-7 cells. The compound inhibited cell growth and triggered apoptosis by affecting the expression of Bax and Bcl-2 in the cells (Zhu et al. 2018). Arenobufagin also reduced viability of MCF-7 cells and induced apoptosis in a time- and dose-dependent manner (Deng et al. 2018).

**Figure 1.**
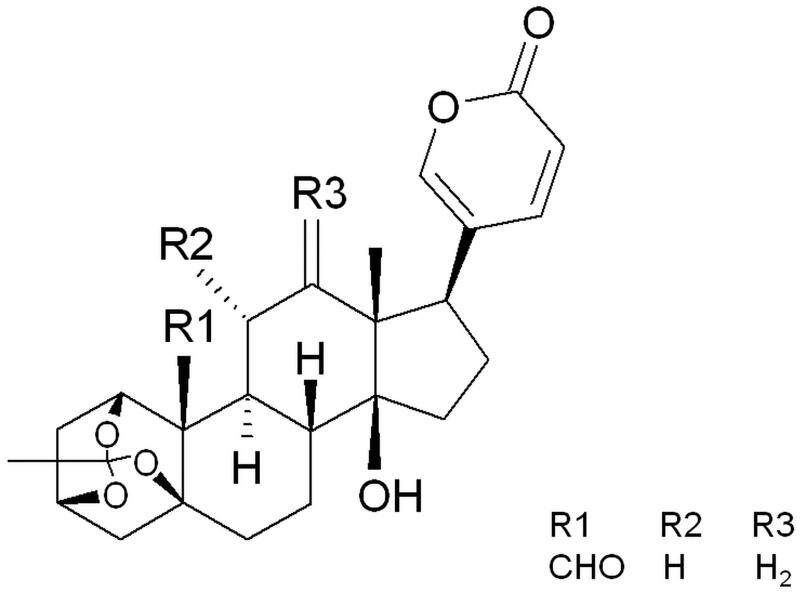
The structure of bersaldegenin-1,3,5-orthoacetate.

Some of the reports describe investigations on *Kalanchoe* species, which are more and more popular as house ornamental plants. These plants have antioxidant, anti-inflammatory, antibacterial, and cytotoxic properties. However, only *K. brasiliensis* Camb. has been described as a remedy in human prostate cancer treatment (Johnson 1999). Studies on cytotoxic activities of *Kalanchoe* plants containing bufadienolides are focussed on main isolated metabolites. For example, bryophyllin B (from *K. pinnata* (Lam.) Pers.) has strong cytotoxic activity on the KB cell line (Yamagishi et al. 1989). Kalanchosides A–C (from *K. gracilis* Hance) revealed toxic activity on lung (A549), prostate (PC-3), epidermoid (A431), and ovarian (1A9) cancer cell lines (Wu et al. 2006). Bufadienolides isolated from the leaves of *K. pinnata* and *K. daigremontiana* × *tubiflora* (Harv.) Raym.-Hamet & H. Perrier showed antitumor-promoting activity on Burkitt’s lymphoma Raji cells (Supratman et al. 2001). Kalantuboside A and B, bryotoxin C, bersaldegenin-1-acetate, and bersaldegenin-1,3,5-orthoacetate from *K. tubiflora* showed significant effects against oral adenosquamous carcinoma (Cal-27), lung adenocarcinoma (A549), promyelocytic leukaemia (HL-60), and melanoma (A2058) cell lines (Huang et al. 2013). In our previous study on *K. daigremontiana*, bersaldegenin-1,3,5-orthoacetate (Figure 1) demonstrated strong cytotoxic activity on cervical (HeLa), ovarian (SKOV-3), melanoma (A375), and breast (MCF-7) cell lines (Stefanowicz-Hajduk et al. 2020). Some of the above mentioned bufadienolides have determined the molecular mode of cell death. However, the pathway of bersaldegenin compounds has not yet been described.

In the present study, we determined the effect of bersaldegenin-1,3,5-orthoacetate on cervical cancer HeLa cells and investigated the role of selected factors in cellular signalling pathways related to cell death.

## Materials and methods

### Isolation of bersaldegenin-1,3,5-orthoacetate from plant material

The method of isolation and structure elucidation of bersaldegenin-1,3,5-orthoacetate was described by Moniuszko-Szajwaj et al. (2016). Briefly, the ground roots of *K. daigremontiana* were extracted with water at room temperature, and concentrated under reduced pressure. Next, the extract was purified on a LiChroprep RP-18 preparative column (100 mm × 60 mm, 40–63 µm) with 80% (v/v) aqueous methanol and the bufadienolide-rich fraction was obtained. This fraction was then partitioned between water and butanol to afford *n*-BuOH extract, which was applied on a Sephadex LH-20 column (950 mm × 22 mm, 25–100 μm), eluted with MeOH, and afforded subfractions, which were separated on a Lichroprep RP-18 column (32 mm × 320 mm, 40–63 μm), eluted with a linear gradient of 15%–40% (v/v) aqueous MeOH in a 0.1% (v/v) HCOOH. Individual bufadienolides were purified isocratically by a semi-preparative HPLC on a C18 column (Kromasil 100-5-C18, 250 mm × 10 mm, 5 μm), using different concentrations of aqueous methanol solutions with 0.1% (v/v) HCOOH. The one from the isolated compounds was bersaldegenin-1,3,5-orthoacetate. The yield of this compound was 0.32 mg (0.032%) in 1 g of dry mass of the root. Its structure elucidation was performed using ESI-MS and NMR spectral data analyses, optical rotation, circular dichroism and IR spectroscopy. The compound was dissolved in sterile DMSO at a concentration 20 mg/mL.

### Cell line culture

The human cervical adenocarcinoma cell line (HeLa S3) was obtained from the American Type Culture Collection (ATCC, Manassas, VA, USA). The cell line was cultured in a humidified 5% CO_2_ incubator (37 °C) in Dulbecco’s Modified Eagle’s Medium (DMEM) supplemented with 10% (v/v) foetal bovine serum (FBS), 100 units/mL of penicillin, and 100 µg/mL of streptomycin (Sigma-Aldrich, St. Louis, MO, USA).

### RTCA cell proliferation assay and Hoechst staining

To monitor HeLa cell viability and proliferation we used the xCELLigence Real-Time Cell Analyzer Dual Plate (RTCA DP, ACEA Biosciences, San Diego, CA, USA) as described in our previous study (Adamska et al. 2019). Briefly, the cells were seeded in E-plates 16 (ACEA Bioscences) at densities of 2 × 10^4^ cells/well. After 24 h, bersaldegenin-1,3,5-orthoacetate was added to the cells at a concentration range of 0.1–20.0 µg/mL for 24 h. Additionally, DMSO – as a solvent of the compound, was added to HeLa cells (a control sample) at a concentration of 0.25% (v/v). Vinblastine sulphate was used as a positive control. The RTCA software v. 1.2.1. calculated IC_50_ values. All the experiments were performed in duplicate, in three independent repeats (n = 6).

To show the effect of bersaldegenin-1,3,5-orthoacetate in HeLa cell nuclei, we stained the cells with the blue fluorescent Hoechst 33342 dye (Life Technologies, Carlsbad, CA, USA). HeLa cells were seeded in 12-well plates at a density of 1 × 10^5^ cells/well. After 24 h, the cells were treated with bersaldegenin-1,3,5-orthoacetate at concentrations of 0.5, 1.0, 2.0, and 5.0 µg/mL for another 24 h. The concentration of the solvent (DMSO) in a control sample was 0.25% (v/v). Then, the cells were stained with the Hoechst dye (1.0 µg/mL) and observed under a fluorescent microscope (Leica, Heerbrugg, Switzerland).

### Assessment of mitochondrial membrane potential (MMP) and reactive oxygen species (ROS) generation

HeLa cells were seeded in 12-well plates at a density of 1 × 10^5^ cells/well and treated with bersaldegenin-1,3,5-orthoacetate at concentrations of 0.1, 0.5, 1.0, 2.0, and 5.0 µg/mL. The concentration of DMSO in a control sample was 0.25% (v/v). After 3 h, 24 h, and 48 h of incubation, the cells were harvested and prepared according with Muse MitoPotential Assay Kit (Merck Millipore, Burlington, MA, USA) protocol. The amount of depolarized/live/dead cells was determined by Muse Cell Analyzer (Merck Millipore). All the experiments were independently repeated three times.

To determine the effect of bersaldegenin-1,3,5-orthoacetate on oxidative stress production in HeLa cells, we treated the cells (1 × 10^5^ cells/well) with the compound at concentrations of 0.1, 0.5, 1.0, 2.0, and 5.0 µg/mL for 24 h. The DMSO concentration in a control sample was 0.25% (v/v). After 24 h of incubation, the cells were stained with Muse Oxidative Stress Kit (Merck Millipore), according with the manufacturer’s protocol. All samples were analysed by Muse Cell Analyzer. The experiments were repeated three times, independently.

### Caspase-3/7/9 activity

The activity of caspase-3/7 was measured according with method described previously (Adamska et al. 2019). Briefly, the cells were seeded in 12-well plates (1 × 10^5^ cells/well) and treated with bersaldegenin-1,3,5-orthoacetate at concentrations of 0.1, 0.5, 1.0, 2.0, and 5.0 µg/mL. The concentration of DMSO added to a control sample did not exceed 0.25% (v/v). After 24 h, the cells were harvested and prepared according with the protocol of Muse Caspase-3/7 Assay Kit (Merck Millipore). Then, the cells were analysed by Muse Cell Analyzer. The experiments were performed at least in three independent repeats.

The activity of caspase-9 was measured by a luminometer. The cells were seeded in 96-well plates and exposed to bersaldegenin-1,3,5-orthoacetate at concentrations of 0.1, 0.5, 1.0, 2.0, and 5.0 µg/mL. The DMSO concentration in a control sample was 0.25% (v/v). The activity of caspase-9 was measured in the cells after 1, 2, 3, 4, 14, and 24 h of incubation the cells with the compound. We used Caspase-Glo 9 Assay Kit (Promega, Madison, WI, USA) and Glomax Multi + Detection System (Promega), according to the manufacturer’s instruction. The experiments were repeated three times, independently.

### Cell cycle analysis

To determine the HeLa cell cycle arrest after treatment with bersaldegenin-1,3,5-orthoacetate, we seeded the cells (5 × 10^5^ cells/well) and incubated with the compound at concentrations of 0.1, 0.5, 1.0, 2.0, and 5.0 µg/mL for 48 h. DMSO concentration in a control sample was 0.25% (v/v). Next, the cells were harvested and stained with Muse Cell Cycle Assay Kit (Merck Millipore), following the manufacturer’s protocol. The amount of HeLa cells in G0/G1, S, and G2/M phases of cell cycle was determined by Muse Cell Analyzer. The experiments were independently repeated three times.

### DNA damage analysis

#### DNA damage analysis with the comet assay

The evaluation of DNA damage was analysed with the alkaline comet assay using the Reagent Kit for Single Cell Gel Electrophoresis Assay (Trevigen, Gaithersburg, MD, USA) according to the manufacturer’s instructions. HeLa cells were seeded at a density of 10^5^ cells/well in 12-well plates. Cells were treated with bersaldegenin-1,3,5-orthoacetate at the concentrations of 1.0 and 5.0 µg/mL for 6 h and 24 h. Next cells were collected and combined with low melting agarose at a ratio of 1:10 and 50 µL of cell suspension was spread onto the CometSlides (Invitrogen/Thermo Fisher Scientific, Waltham, MA, USA). Slides were immersed in the lysis solution for 1 h at 4 °C, after which they were placed in the alkaline unwinding solution for 1 h at 4 °C. Slides were next placed in the electrophoresis slide tray and immersed in the alkaline electrophoresis solution in the CometAssay ES unit (Trevigen). Slides were submitted to electrophoresis at 1 V/cm for 30 min. Following electrophoresis, slides were washed and then stained with Sybr Gold (Invitrogen/Thermo Fisher Scientific, Waltham, MA, USA) for 30 min at RT. Slides were analysed under a fluorescence microscope (Nikon PCM-2000) and images of at least 50 comets per slide were analysed with the TriTek CometScore 2.0 software. Two regions of the comet were selected, the whole cellular DNA and the region of DNA within the comet head. The densities of the regions were defined and the results are presented as tail moment expressed as the percentage of DNA in the tail multiplied by the tail length. Each treatment was normalized to that of the control.

#### Immunofluorescence staining

HeLa cells were seeded in a 6-well plate with coverslips (5 × 10^5^ cells/well) and treated with bersaldegenin-1,3,5-orthoacetate at concentrations of 1 and 5 µg/mL. DMSO was added to the cells (control) at a concentration of 0.25% (v/v). A positive control was etoposide added to the cells at concentration of 10 μM. After 6 h and 24 h, the cells on the coverslips were fixed with 4% formaldehyde (v/v) at room temperature for 10 min and then, permeabilized with 0.1% Triton-X100 (Sigma-Aldrich) for 15 min. Blocking step was performed with 5% bovine serum albumin (BSA, w/v) for 1 h. Subsequently, the cells were incubated with the anti-phospho-H2A.X (Ser139), clone JBW30, Alexa Fluor 555 Conjugate antibody (1:100, mouse monoclonal, Merck Millipore) in 1% BSA (w/v) for 1 h. After three steps of washes, the cells on the coverslips were incubated with Hoechst 33342 dye (2.0 µg/mL) at room temperature for 20 min and observed under a fluorescence microscope. Images of at least 100 nuclei per slide were analysed.

### Gene expression analysis

We seeded HeLa cells (3 × 10^6^ cells/well) in 6-well plates and treated with bersaldegenin-1,3,5-orthoacetate at a concentration of 1.0 μg/mL for 24 h. The control sample was treated with DMSO at a concentration 0.05% (v/v). Next, the cells were harvested and the isolation of total RNA was performed with RNeasy Mini Kit (Qiagen, Venlo, The Netherlands) according with the manufacturer’s instruction. The quality and concentration of isolated RNA was measured by Agilent Technologies 4200 TapeStation (Agilent Technologies, Santa Clara, CA, USA), according with the manufacturer’s protocol. Next, we performed synthesis of cDNA using Maxima First Strand cDNA Synthesis Kit (Thermo Fisher Scientific, Waltham, MA, USA), following with the manufacturer’s protocol. Obtained cDNA was applied on the Applied Biosystems TaqMan Array Human Apoptosis 96-well FAST Plates (Life Technologies, USA) that contain 92 assays for cell death associated genes and 4 assays for endogenous control genes (18S, GAPDH, HPRT1, and GUSB) (Table S1). The PCR reactions were performed on StepOnePlus Real-Time PCR System (Life Technologies). All the obtained data were analysed by StepOne software v.2.3. The experiments were performed three times, independently.

### Statistical analysis

Statistical data were analysed using the STATISTICA 12.0 software package (StatSoft. Inc., Tulsa, OK, USA). All data are expressed as mean values ± standard deviation (SD). For comparison studies, Student’s *t*-test was performed. The statistical significance was set at *p* < 0.05.

## Results

### Bersaldegenin-1,3,5-orthoacetate showed strong cytotoxic activity in HeLa cells

**Figure 2.**
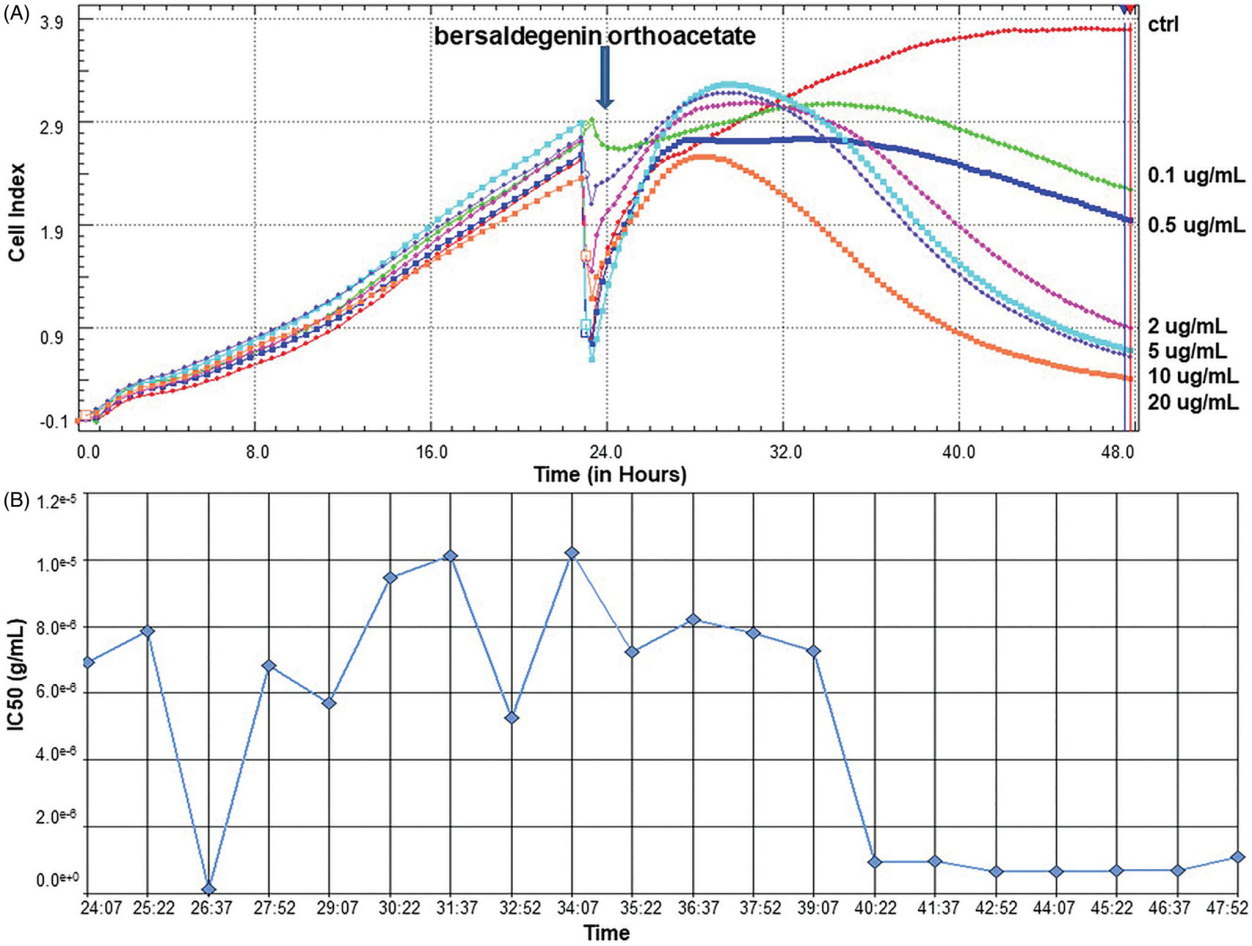
The RTCA viability profiles of HeLa cells treated with bersaldegenin-1,3,5-orthoacetate. The cells were treated with the compound in a concentration range of 0.1–20.0 µg/mL for 24 h. DMSO was added to the cells (a control sample) at a concentration of 0.25% (v/v). (A) The profiles of HeLa cells proliferation, (B) IC_50_ values calculated during 24 h of treatment the cells with the compound. All the profiles were obtained by RTCA software v.1.2.1.

HeLa cells were treated with the compound for 24 h and the obtained results clearly indicate that bersaldegenin is a strong inducer of the cell death. The monitoring of cell behaviour in continuous and real-time manner by RTCA system allowed to observe the reduction of HeLa cells viability after treatment with the compound. The concentrations of bersaldegenin above 0.1 µg/mL caused decrease in the cellular profiles on RTCA graph (Figure 2). The IC_50_ value of bersaldegenin-1,3,5-orthoacetate (0.55 µg/mL), calculated in our previous study (Stefanowicz-Hajduk et al. 2020), indicated significant cytotoxic effect on HeLa cells. This effect changed with the compound dose and incubation time (Figure 2(B)). The IC_50_ value of vinblastine sulphate, used as a positive control, was 4.55^−03 ^µg/mL.

**Figure 3.**
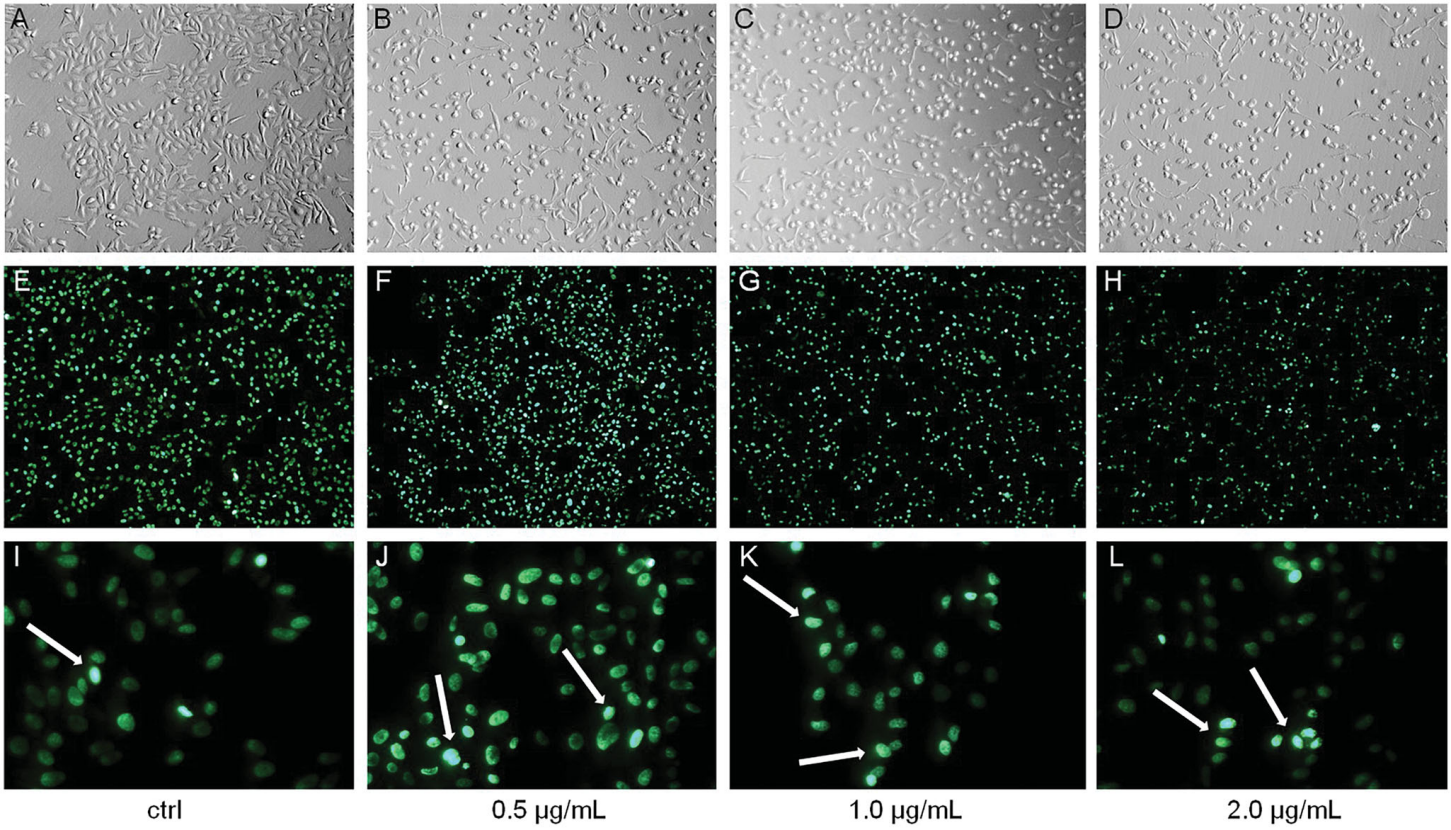
The nuclei of HeLa cells stained with Hoechst 33342 dye after 24 h of treatment with bersaldegenin-1,3,5-orthoacetate. (A–D) show the cells under an inverted light microscope, (E–L) – under a fluorescent microscope. The cells were treated with DMSO (0.25%) – a control sample (A, E, I) and the bersaldegenin at concentrations of 0.5 (B, F, J), 1.0 (C, G, K), and 2.0 μg/mL (D, H, L). White arrows indicate Hoechst strongly positive nuclei. The images were done at 100× (A–H) and 400× magnification (I–L), respectively.

Additionally, we observed the effect of bersaldegenin-1,3,5-orthoacetate in HeLa cells nuclei after 24 h of treatment. The cells were stained with Hoechst 33342 dye, which emits fluorescence when bounds to DNA. The treated HeLa cell nuclei mostly showed homogenous dye lighting without clear dye aggregation in comparison to the control cells (Figure 3).

### Bersaldegenin-1,3,5-orthoacetate increased the level of cellular oxidative stress

**Figure 4.**
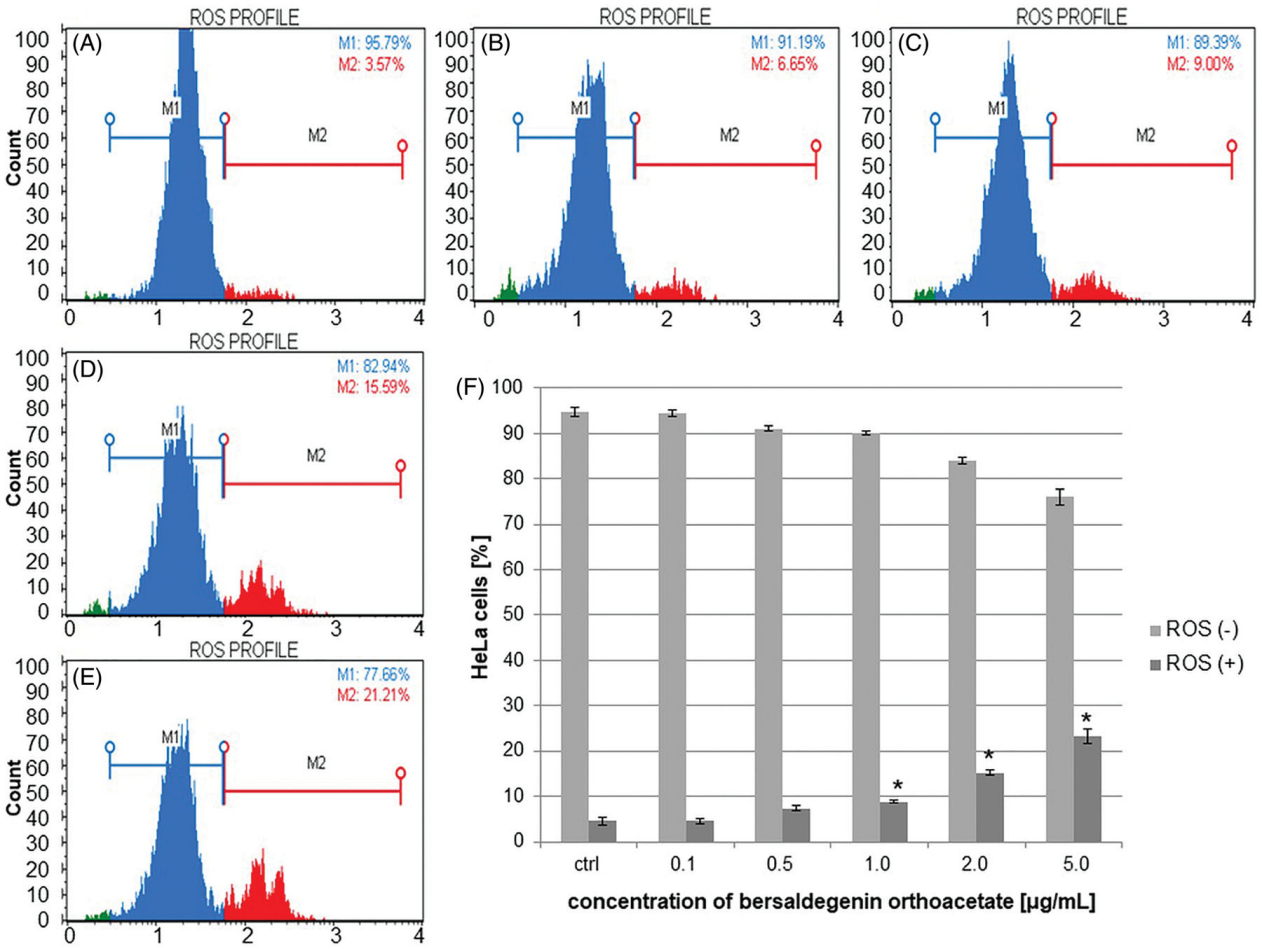
The effect of bersaldegenin-1,3,5-orthoacetate on ROS generation in HeLa cells. The cells were treated with the compound at concentrations of 0.1–5.0 µg/mL. The ROS negative (ROS−) and ROS positive (ROS+) cells were assessed by flow cytometry and determined in comparison to the control sample (0.25% DMSO). A–E show ROS level in HeLa cells treated with DMSO (A, the control) and the bersaldegenin at concentrations of 0.5 (B), 1.0 (C), 2.0 (D), and 5.0 µg/mL (E). (F) – presents the percentage of each cell population after 24 h of treatment the cells with the compound. Each sample was run in triplicate. Error bars indicate standard deviations. Significant differences relative to the control are marked with an ‘*’ (*p* < 0.05).

To estimate the effect of bersaldegenin-1,3,5-orthoacetate on reactive oxygen species (ROS) generation in HeLa cells, we treated the cells with different concentrations of the compound. After 24 h, we analysed the cells by flow cytometry and observed significant increase in amount of ROS positive cells above the compound concentration of 1.0 µg/mL. The percentage of ROS (+) cells were 4.52 ± 0.5, 7.39 ± 0.59, 8.84 ± 0.22, 15.18 ± 0.55, 23.21 ± 1.68% for 0.1, 0.5, 1.0, 2.0, and 5.0 µg/mL, respectively (Figure 4).

### Bersaldegenin-1,3,5-orthoacetate did not modulate MMP (ΔΨm) in HeLa cells

**Figure 5.**
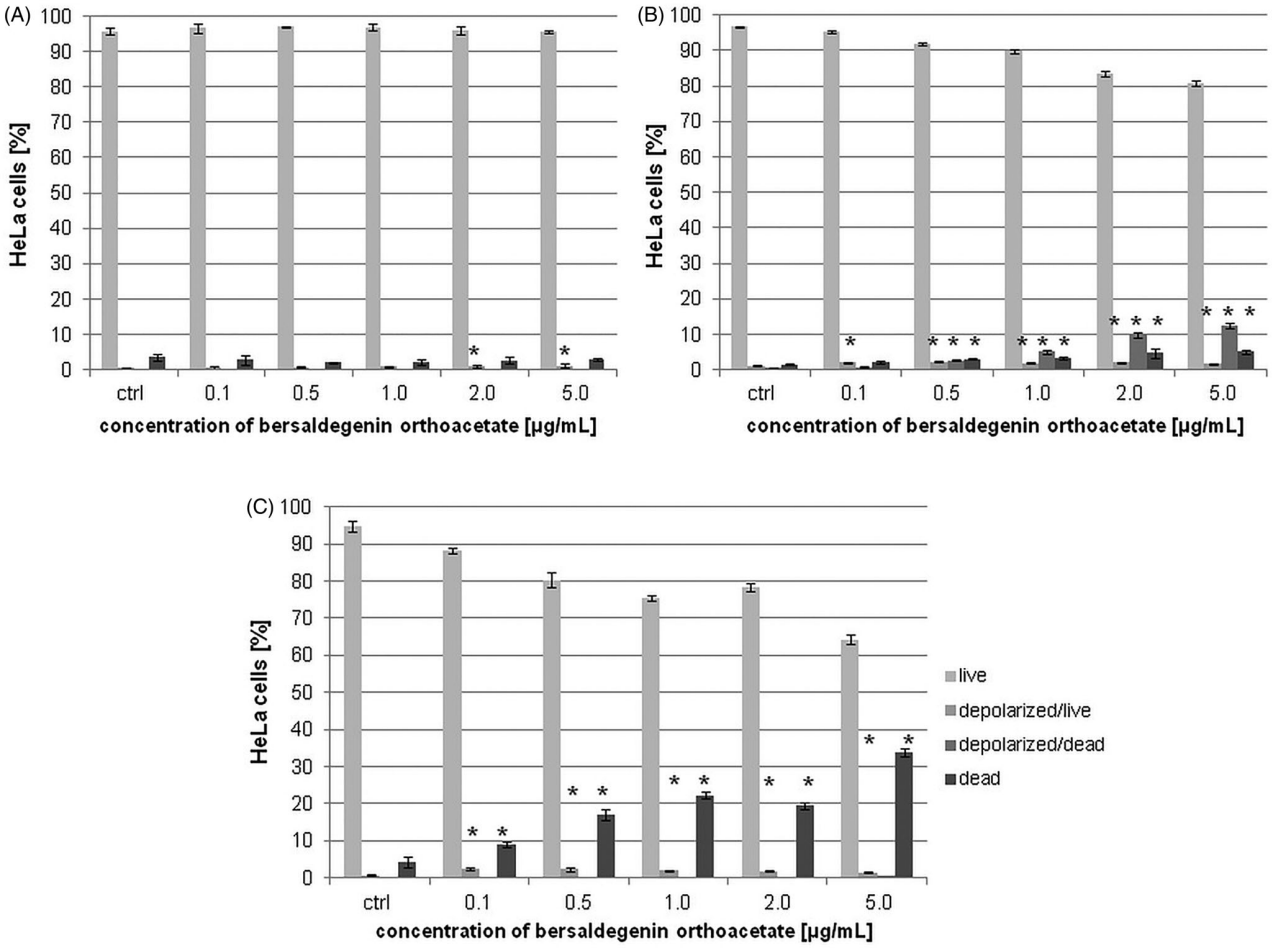
The effect of bersaldegenin-1,3,5-orthoacetate on MMP in HeLa cells. The cells were treated with the compound at concentrations 0.1–5.0 µg/mL and DMSO (a control sample). After 3 h (A), 24 h (B), and 48 h (C) the cells were stained and analysed by flow cytometry. The percentage of live, depolarized/live, depolarized/dead and dead cells was determined in comparison to the control sample. Each sample was run in triplicate. Error bars represent standard deviations. Significant differences relative to the control are marked with an asterisk ‘*’ (*p* < 0.05).

To study the effect of the compound on the mitochondrial membrane potential (MMP) in HeLa cells, we treated the cells with different concentrations of the compound (0.1–5.0 µg/mL). The obtained results indicate that bersaldegenin did not significantly affect MMP in living cells. We observed depolarization only in the dead cells after 24 h of incubation. The percentage of depolarized/dead cells was 0.67 ± 0.16, 2.7 ± 0.22, 5.02 ± 0.43, 9.89 ± 0.71, 12.41 ± 0.69% at the compound concentrations of 0.1, 0.5, 1.0, 2.0, and 5.0 µg/mL, respectively. After 48 h, the percentage of depolarized/dead cells was comparable to the control. Then, in the amount of depolarized/live cells we observed slight increase (from 0.81 ± 0.24 for the control to 2.51 ± 0.32% for the bersaldegenin concentration of 0.1 µg/mL). After 3 h, we did not observe any changes in decrease in mitochondrial potential in the living and dead cells after treatment with the compound in comparison to the control sample (Figure 5).

### Caspases-3/7/9 were not activated by bersaldegenin-1,3,5-orthoacetate

**Figure 6.**
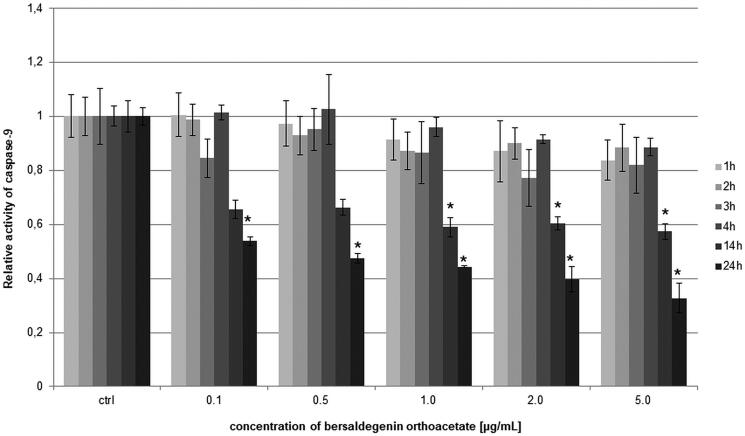
The relative activity of caspase-9 in HeLa cells after treatment with bersaldegenin-1,3,5-orthoacetate. The cells were incubated with the compound at concentrations of 0.1–5.0 µg/mL for 1–24 h. The caspase activity was estimated by luminometer and is presented in comparison to the control (0.25% DMSO). Each sample was run in triplicate. Error bars indicate standard deviations. Significant differences relative to the control are marked with an ‘*’ (*p* < 0.05).

The activity of caspase-9 was measured in HeLa cells after treatment with bersaldegenin-1,3,5-orthoacetate for 1, 2, 3, 4, 14 and 24 h. The caspase-9 activity estimated by luminometric method revealed that the tested compound did not increase the activity level of this caspase in the cells in all the measurement points in comparison to the control. Furthermore, we observed significant decrease in the activity of caspase-9 after 24 h of treatment the cells with the compound (Figure 6).

**Figure 7.**
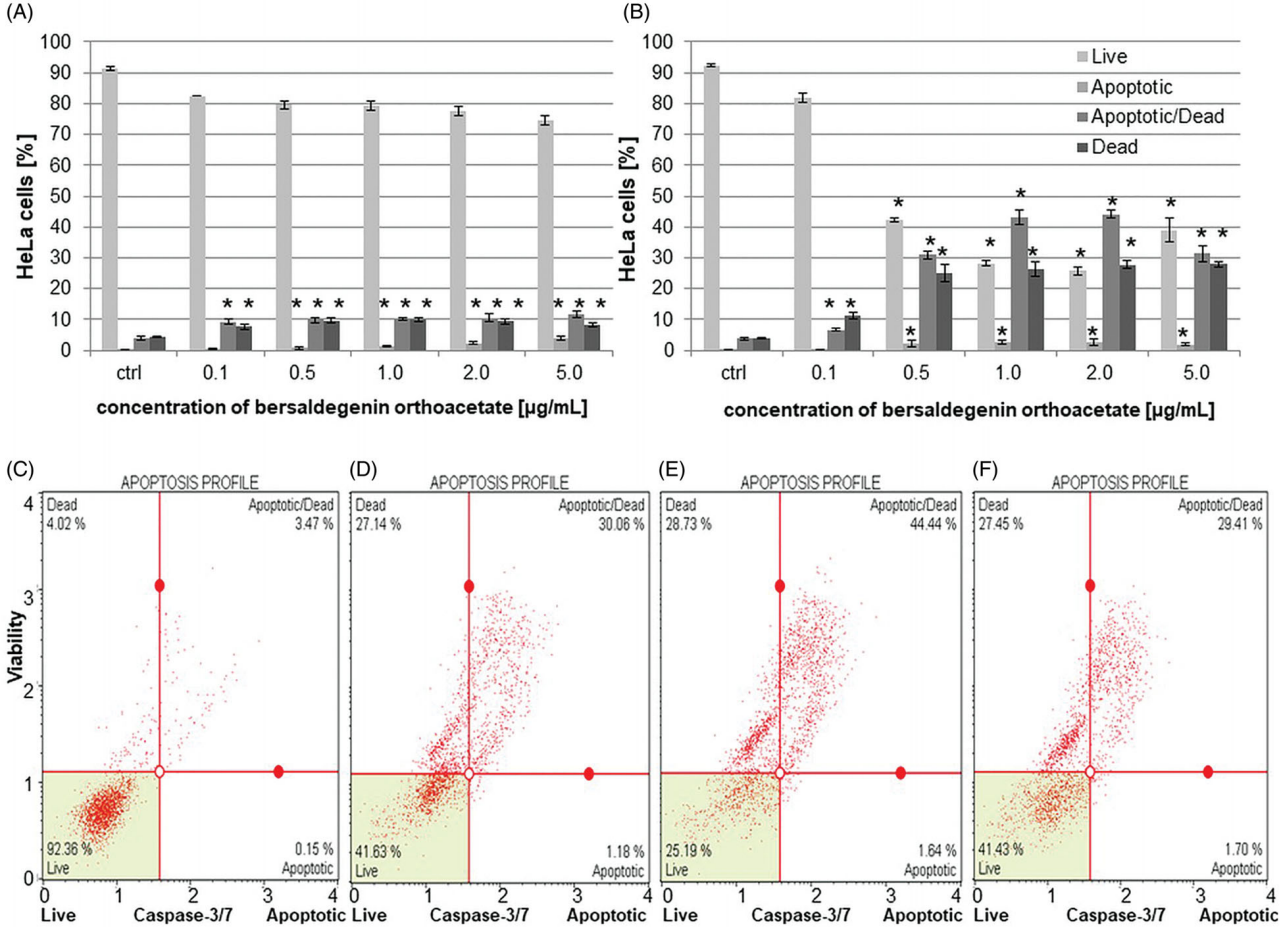
The activity of caspase-3/7 in HeLa cells after treatment with bersaldegenin-1,3,5-orthoacetate. The cells were treated with the compound at concentrations of 0.1–5.0 µg/mL for 24 h and 48 h. The caspase activity was estimated by flow cytometry and the graph presented live, apoptotic, and dead cell populations in comparison to the control (0.25% DMSO) after 24 h (A) and 48 h (B) of treatment. C–F present the effect of DMSO (C) and the compound (0.5 (D), 2.0 (E), 5.0 µg/mL (F), respectively) on HeLa cells after 48 h of treatment. Each sample was run in triplicate. Error bars indicate standard deviations. Significant differences relative to the control are marked with an ‘*’ (*p* < 0.05).

Furthermore, the activity of caspase-3/7 is indicated by an increase in population of early apoptotic cells. In the treated cells with the compound after 24 h and 48 h, the activity of caspase-3/7 was slightly higher than in the untreated cells. Also, we observed a significant increase in the population of late apoptotic and dead cells what indicated that the main pathway of the HeLa cell death is caspase-3/7 independent (Figure 7).

### Bersaldegenin-1,3,5-orthoacetate induced DNA damage in HeLa cells

**Figure 8.**
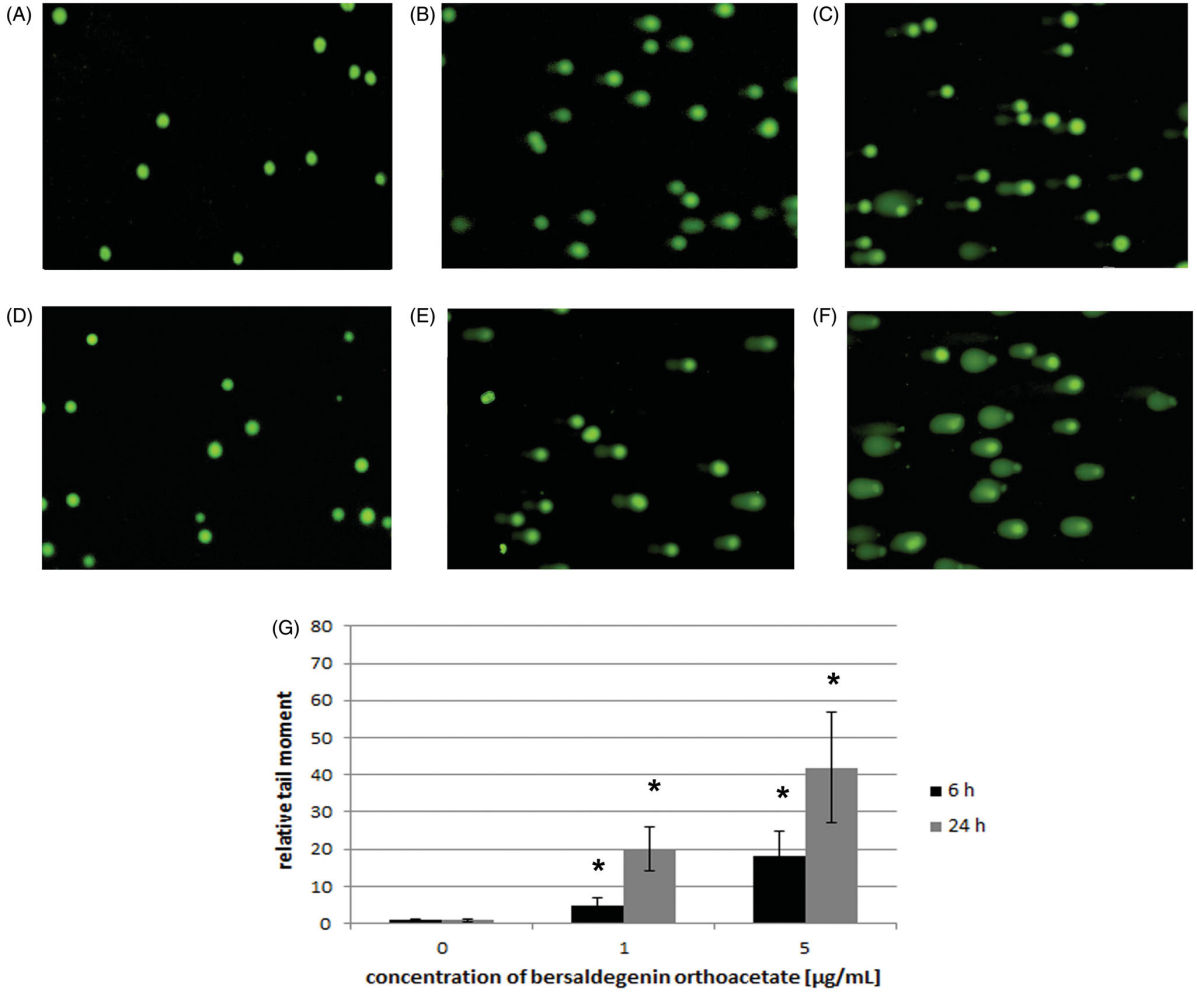
Induction of DNA damage by bersaldegenin-1,3,5-orthoacetate in HeLa cells. Cells were treated with concentrations of 1.0 and 5.0 μg/mL for 6 and 24 h. The extent of DNA damage was compared to control cells. (A–F) represent images of DNA damage induced in HeLa cells treated with DMSO (A) and the compound 1.0 µg/mL (B), 5.0 µg/mL (C) for 6 h and DMSO (D) and the compound 1.0 µg/mL (E), 5.0 µg/mL (F) for 24 h. DNA damage was analysed with the comet assay and defined as tail moment (G). Results are means of (± SD) of three repetitions. Significant differences between control and treated samples are indicated with an ‘*’ (*p* < 0.05).

The effects of bersaldegenin-1,3,5-orthoacetate on the induction of DNA damage in HeLa cells were evaluated with the alkaline comet assay. The effects of the compound at the concentrations of 1.0 and 5.0 µg/mL were evaluated after 6 h and 24 h of treatment. The degree of DNA damage was determined by defining the extent of DNA migration in the comet and results are presented as tail moment, which is the product of the percentage of total DNA in the tail and tail length. The results of the comet assay revealed increase in DNA damage as determined by the increase in tail moment. After 6 h of treatment, 5-fold and 18-fold increases in DNA damage were observed, as compared to control samples, at the concentrations of bersaldegenin-1,3,5-orthoacetate 1.0 and 5.0 µg/mL, respectively. At longer time treatment, a further increase in DNA damage was observed, and the tail moment increased 20-fold and 42-fold at the concentrations of 1.0 and 5.0 µg/mL, respectively (Figure 8).

**Figure 9.**
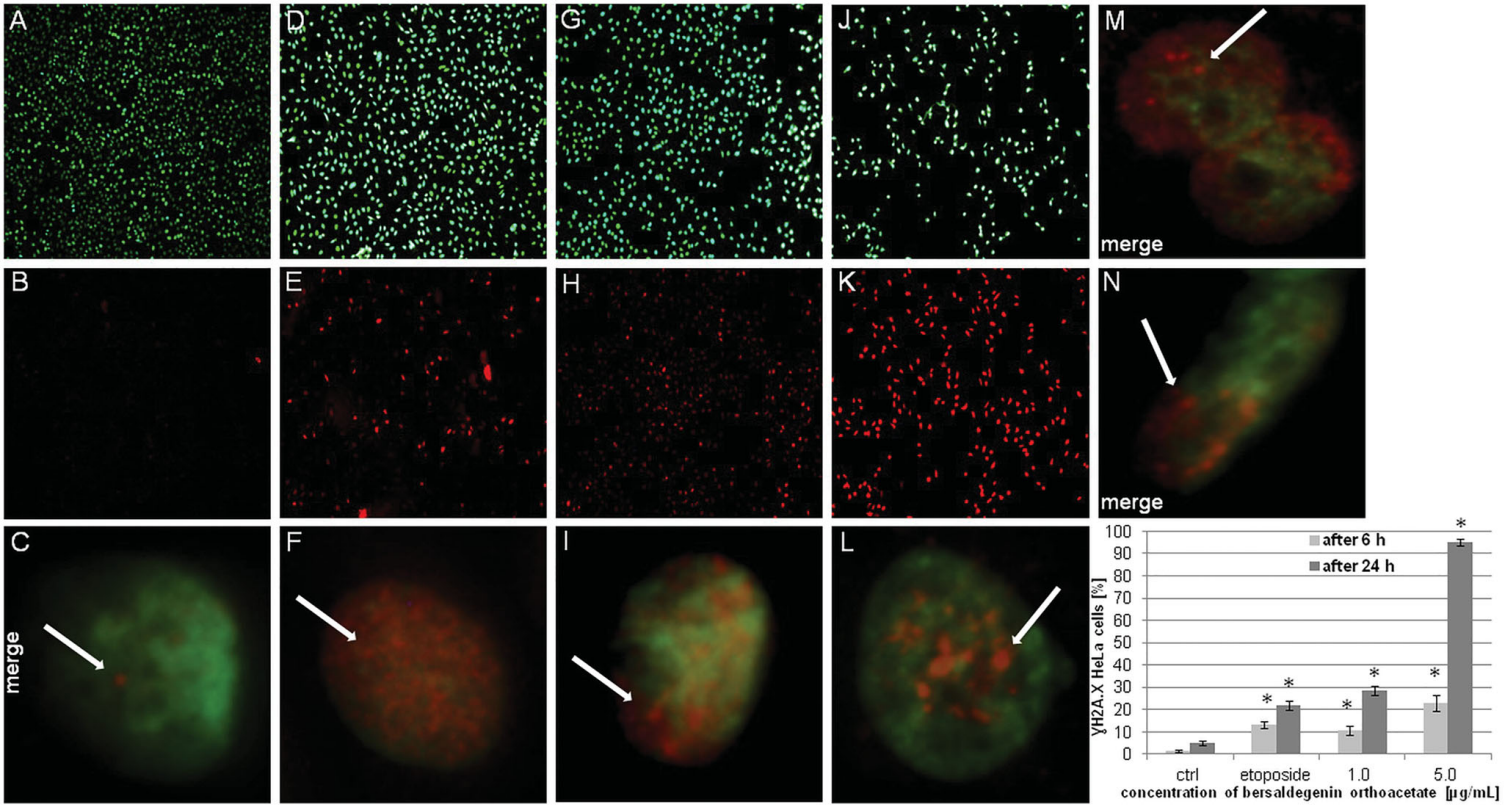
Bersaldegenin-1,3,5-orthoacetate induced phosphorylation of H2A.X in HeLa cells nuclei. DMSO (0.25% v/v, a negative control, A–C) and etoposide (10 μM, a positive control, D–F), and the compound were added to the cells at concentrations of 1.0 (G–I) and 5.0 μg/mL (J–L) for 24 h. (M) and (N) show HeLa nuclei after 6 h of incubation with the bersaldegenin compound at concentrations of 1 μg/mL (M) and 5 μg/mL (N). The nuclei were stained with Hoechst 33342 dye (A, D, G, J, M, N) and anti-phospho-H2A.X (Ser139) Alexa Fluor 555 conjugate antibody (B, E, H, K, M, N). Arrows indicate γH2A.X foci signals visible in the nuclei. The graph shows the γH2A.X nuclei (%) with high fluorescence signal in the controls and the treated HeLa cells. The experiment was repeated twice. Error bars indicate standard deviations. Significant differences relative to the control are marked with an ‘*’ (*p* < 0.05). The images were done with a fluorescence microscope at 100× and 630× magnification.

Additionally, to confirm induction of DNA damage by bersaldegenin orthoacetate in HeLa cells we performed immunofluorescence staining. The cells were treated with the compound at concentrations of 1.0 and 5.0 μg/mL for 6 h and 24 h. The obtained results showed that the amount of the cells with phospho histone H2A.X (γH2AX) increased after treatment with the compound and γH2AX signal was higher in these nuclei than in DMSO control (Figure 9). Most of the treated cells expressed γH2AX signal that was distributed throughout the nuclei. Therefore, the compound-induced γH2AX could not be quantified by the direct visual counting of ‘foci’ and the activation is presented as the percentage of γH2AX positive nuclei in the HeLa cells populations.

### Bersaldegenin-1,3,5-orthoacetate induced arrest of HeLa cell cycle in G2/M phase

**Figure 10.**
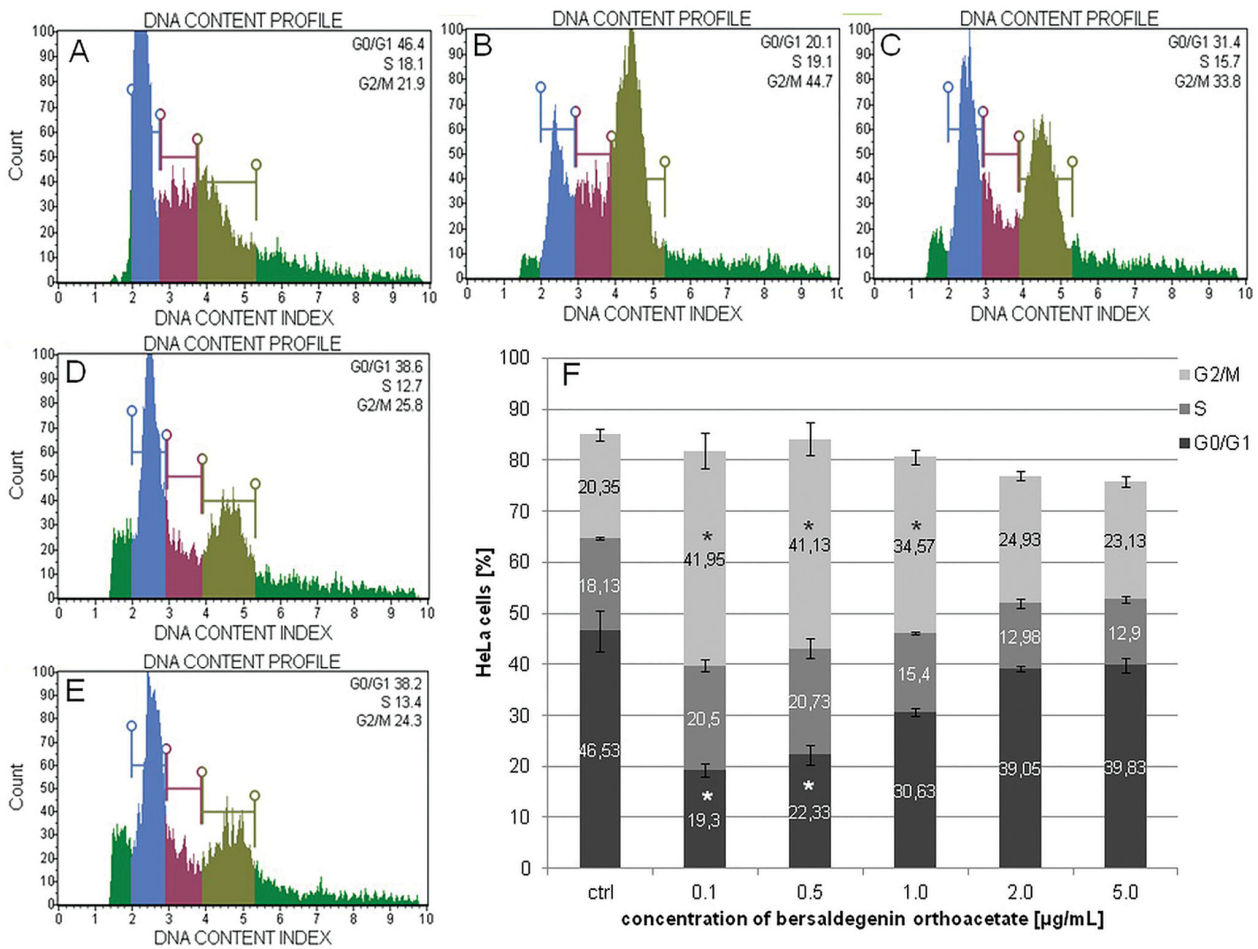
The effect of bersaldegenin-1,3,5-orthoacetate on cell cycle in HeLa cells after 48 h of treatment. The cells were treated with DMSO (A) and the compound at concentrations of 0.1-5.0 μg/mL (0.5 (B), 1.0 (C), 2.0 (D), and 5.0 µg/mL (E), respectively) and analysed by flow cytometry. The percentage of the cells in each phase was determined in comparison to the control (0.25% DMSO) (F). The experiment was repeated three times, independently. Error bars represent standard deviations. Significant differences relative to the control are marked with an asterisk ‘*’ (*p* < 0.05).

After 48 h of treatment the cells with the compound, we estimated cell cycle distribution by flow cytometry. The obtained results indicate that bersaldegenin significantly arrested cell cycle in G2/M phase and slightly in S phase (Figure 10). The highest changes in inhibition of G2/M phase were observed for the compound concentrations of 0.1, 0.5, and 1.0 μg/mL. The percentage of the cells in G2/M phase was 41.95 ± 3.48, 41.13 ± 3.11, 34.57 ± 1.42, 24.93 ± 0.84, 23.13 ± 1.09% for 0.1, 0.5, 1.0, 2.0, and 5.0 μg/mL, respectively. We also observed slightly higher amount of the cells in S phase for the compound concentrations of 0.1 and 0.5 μg/mL in comparison to the control. The results were 20.5 ± 1.13 and 20.73 ± 1.96%, respectively.

### Bersaldegenin-1,3,5-orthoacetate induced increase in expression of NF-Kappa-B inhibitors and TNFRSF10, TNFRSF10B genes

**Figure 11.**
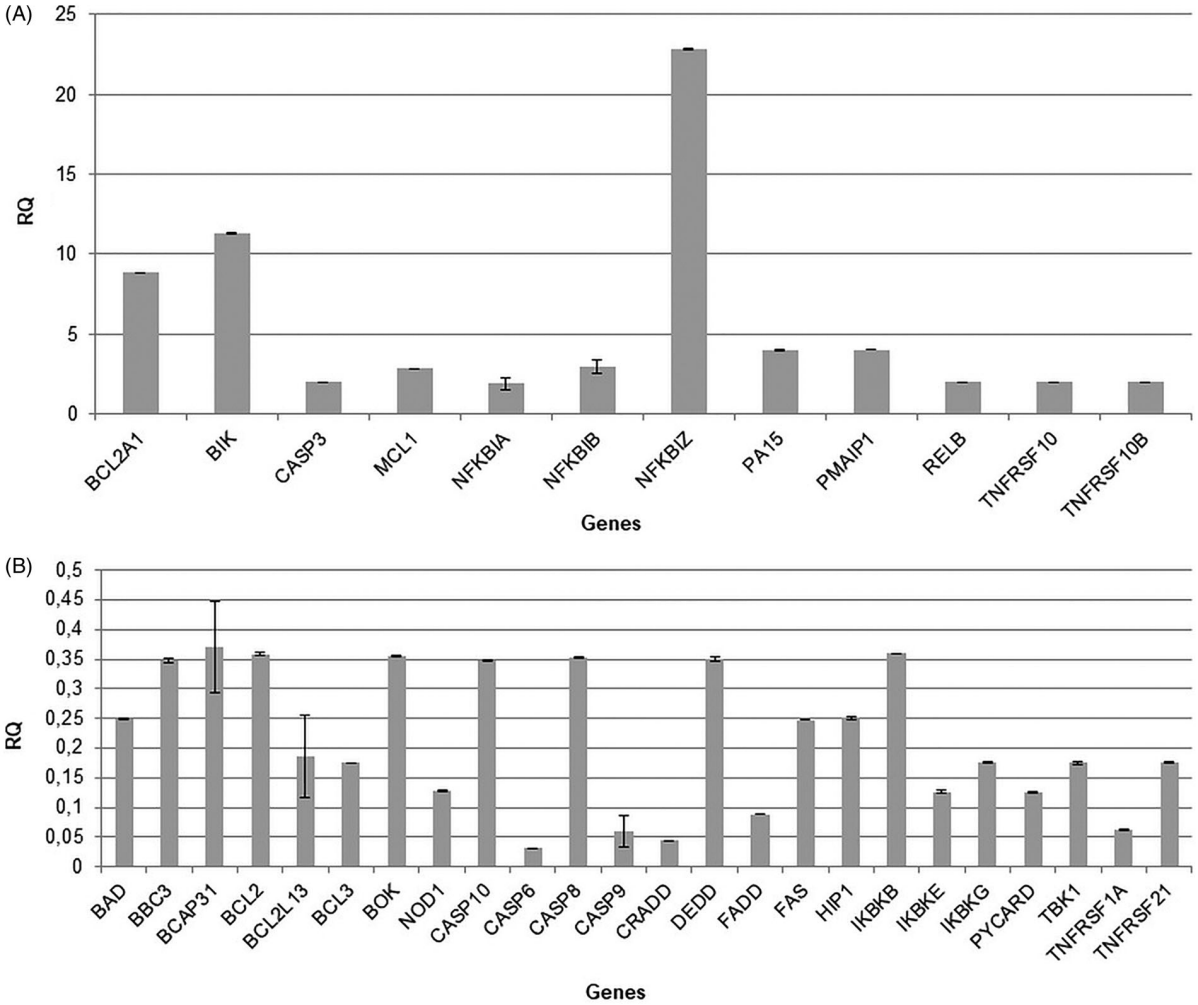
Bersaldegenin-1,3,5-orthoacetate induced changes in the expression of genes in HeLa cells. The cells were treated with 0.05% DMSO (control) and the compound at a concentration of 1.0 µg/mL for 24 h. The expression of genes was normalized to GUSB endogenous control gene and their levels are presented as a fold-change over (A) or under (B) the value 1.0 (ctrl).

The analysis of genes expression in HeLa cells treated with the compound (1.0 μg/mL) showed that the higher expression was observed for BCL2A1 (Bcl-2-Related Protein A1), BIK (Bcl-2-Interacting Killer), CASP3 (Caspase 3, Apoptosis-Related Cysteine Protease), MCL1 (Myeloid Cell Leukaemia Sequence 1), NFKBIA (NF-Kappa-B Inhibitor Alpha), NFKBIB (NF-Kappa-B Inhibitor Beta), NFKBIZ (NF-Kappa-B Inhibitor Zeta), PA15, PMAIP1 (Phorbol-12-Myristate-13-Acetate-Induced Protein 1), RELB (RELB Proto-Oncogene, NF-KB Subunit), TNFRSF10 (Tumor Necrosis Factor Receptor Superfamily Member 10), and TNFRSF10B (Tumor Necrosis Factor Receptor Superfamily, Member 10 b) genes. The expression of these genes was at least two times higher in comparison to the control sample (0.05% DMSO). The genes – BCL2A1, BIK, and NFKBIZ were the most up-regulated. We also observed down-regulation for 24 genes which expression was at least two times lower than in the control sample (Figure 11). The genes – CASP6 (Caspase 6), CASP9 (Caspase 9), NOD1 (Nucleotide Binding Oligomerization Domain Containing 1), CRADD (CASP2 and RIPK1 Domain Containing Adaptor with Death Domain), FADD (Fas-Associating Death Domain-Containing Protein), TNFRSF1A (Tumor Necrosis Factor Receptor Superfamily Member 1 A), IKBKE (Inhibitor of Nuclear Factor Kappa B Kinase Subunit Epsilon), and PYCARD (Caspase Recruitment Domain-Containing Protein 5) were the most down-regulated.

## Discussion

In this study, we estimated the cytotoxic activity of bersaldegenin-1,3,5-orthoacetate on HeLa cells with determination of selected factors that play a role in the cell death pathway. Our results indicate that the compound is strong cytotoxic agent and its effect is time- and dose-dependent.

The antiproliferative and cytotoxic effect of bufadienolides is known, however available data concerning these activities are limited. Han et al. tested a few bufadienolides (hellebrigenin, hellebrigenol, arenobufagin, bufotalin, and bufalin) on human liver cancer cells (HepG2). The results indicated that these compounds have significant antiproliferative effects in a concentration range below 150 ng/mL (Han et al. 2016). These authors also studied arenobufagin and hellebrigenin on human glioblastoma cell line U-87 (Han et al. 2018). Dose-dependent cytotoxicity was observed in these cells at the compounds concentration of about 20 ng/mL. Furthermore, this effect was not observed in normal cells – mouse primary astrocytes. Another authors showed inhibitory effect of bufalin and cinobufagin on the proliferation of androgen dependent (LNCaP) and independent prostate cancer cell lines (DU145 and PC3) (Yeh et al. 2003).

There are only a few reports on biological activity of bersaldegenin derivatives on normal and cancer cells (Kolodziejczyk-Czepas et al. 2016; Stefanowicz-Hajduk et al. 2020). In our previous study, we showed that bersaldegenin-1,3,5-orthoacetate caused very significant decrease in viability of HeLa, SKOV-3, MCF-7, and A375 cell lines. We also observed that this compound did not trigger an increase in the population of early apoptotic cells (Stefanowicz-Hajduk et al. 2020). To estimate the signalling pathway leading to cell death after treatment with the bersaldegenin compound, we have prepared experiments with HeLa cells in the present study. The microscopic, flow cytometry, luminometric, and RT-qPCR results indicate that the tested compound induced mostly caspase-independent cell death. Microscopic observation of the treated cells revealed that the HeLa cell nuclei had disorganized chromatin with homogenous dispersed DNA fragments. These changes, revealed after Hoechst staining, were not similar to typical morphological features of apoptotic cells. Furthermore, another performed experiments showed that bersaldegenin-1,3,5-orthoacetate induced reactive oxygen species overproduction and did not cause depolarization of mitochondrial membrane as well as activation of initiating caspase-9 and effector caspases-3/7. What is important, the compound strongly induced cell cycle arrest in G2/M phase. This is often associated with activation of S- and G2/M phase DNA damage checkpoints. This damage can be induced by exogenous (e.g., alkylating compounds, antitumor chemotherapeutics, plant metabolites, UV, radiation) and endogenous agents such as reactive oxygen and nitrogen species that are produced during cellular metabolism (Jackson & Bartek 2009). In this process, cell cycle arrest and inhibition of DNA replication occur (Mannuss et al. 2012). In our study, we observed double-stranded DNA (dsDNA) damage in HeLa cells treated with bersaldegenin-1,3,5-orthoacetate during the comet assay. Furthermore, as confirmation of the comet assay results we prepared the immunofluorescence staining and observed accumulation of phosphorylated histone H2A.X (γH2AX) in the cells – an indicator of the level of ds DNA damage in nuclei, what also confirms the role of DNA-damage response in the compound treated cells (Jackson & Bartek 2009).

DNA damage plays a crucial role in cell homeostasis and is associated with NF-kappa-B (NF-кB) family factors which regulate physiological and pathological processes. These proteins are located in the cytoplasm and are associated with a family of inhibitors of NF-кB proteins (IкBs). After stimulation, IкB undergoes degradation in a proteasome, and the free NF-кB translocates to the nucleus and regulates genes transcription (Wang et al. 2017). NF-кB activation depends on activity of these inhibitors and can occur in response to DNA damage. Induction of synthesis of NF-кB inhibitors antagonises NF-кB activity and prevents prolonged NF-кB activation (Sun et al. 1993; Chiao et al. 1994). In our study, we show that DNA was damaged in HeLa cells treated with the bersaldegenin and this may be associated with NF-кB pathway. The obtained gene analysis revealed very significant overexpression of NF-кB inhibitors genes (NFκBIZ, NFκBIB, and NFκBIA) and downregulation of IкB kinases family genes. However, further studies evaluating the role of these factors in the cell death pathway need to be performed.

In bufadienolide compounds family, determination of cellular mechanisms leading to cell death was done with cinobufagin. The compound was tested on human multiple myeloma (MM) U266 cells and caused pro-apoptotic effects through ROS-mediated activation of ERK, JNK, and p38 MAPK pathways and the activation of caspase-3 in the cells (Baek et al. 2015). Also in other studies, cinobufagin caused ROS overproduction, decreased MMP in non-small cell lung cancer (NSCLC) (Zhang et al. 2016). The compound induced cell cycle arrest and apoptosis by activation of caspase-3, chromatin condensation, DNA degradation and inhibition of the AKT/mTOR signalling pathway in the tested cells. Next, gamabufotalin induced autophagy and activation of p38 MAPK pathway in human glioblastoma cell line U-87 (Yuan et al. 2019). Bufalin induced ROS overproduction, reduced MMP, activated caspases-9/-3 and DNA damage in human lung cancer cell line NCI-H460 (Wu et al. 2017).

## Conclusions

In conclusion, bersaldegenin-1,3,5-orthoacetate strongly induces caspase-independent cell death in cancer HeLa cells which is associated with cell cycle arrest in G2/Mphase, ROS overproduction, and double-stranded DNA damage.

## Supplementary Material

Supplemental Material
