# Supplementary material for: Bersaldegenin-1,3,5-orthoacetate induces caspase-independent cell death, DNA damage and cell cycle arrest in human cervical cancer HeLa cells
Source: Pharm Biol. 2021 Jan 6;59(1):54–65. doi: 10.1080/13880209.2020.1866025 (PMC7801116; doi:10.1080/13880209.2020.1866025)
Supplement: Supplemental Material [file IPHB_A_1866025_SM4043.docx]

Supplementary Material

**Table S1.** Symbols of genes from TaqMan Array Human Apoptosis 96-well FAST Plates.

| GENE SYMBOL | | | | | |
| --- | --- | --- | --- | --- | --- |
| 18S (control gene) | GAPDH (control gene) | HPRT1 (control gene) | GUSB (control gene) | BIRC2 | APAF1 |
| BCL2 | BCL2A1 | BCL2L1 | BCL2L10 | BCL2L11 | BCL2L13 |
| BIRC3 | XIAP | BIRC5,EPR1 | BIRC6 | BIRC7 | BIRC8 |
| CARD9 | CASP1 | CASP10 | CASP14 | CASP2 | CASP3 |
| CASP9 | CFLAR | CHUK | CRADD | DAPK1 | DEDD |
| HIP1 | HRK | HTRA2 | CARD18 | IKBKB | IKBKE |
| NFKB1 | NFKB2 | NFKBIA | NFKBIB | NFKBIE | NFKBIZ |
| RIPK1 | RIPK2 | TBK1 | TNF | TNFRSF10A | TNFRSF10B |
| BAD | BAK1 | BAX | BBC3 | BCAP31 | BCL10 |
| BCL2L14 | BCL2L2 | BCL3 | BID | BIK | NAIP |
| BNIP3 | BNIP3L | BOK | NOD2 | NOD1 | CARD6 |
| CASP4 | CASP5 | CASP6 | CASP7 | CASP8 | CASP8AP2 |
| DEDD2 | DIABLO | IFT57 | FADD | FAS | FASLG |
| IKBKG | LRDD | LTA | LTB | MCL1 | NLRP1 |
| PEA15 | PMAIP1 | PYCARD | REL | RELA | RELB |
| TNFRSF1A | TNFRSF1B | TNFRSF21 | TNFRSF25 | TNFSF10 | TRADD |
